# Programmed cell death pathways coordinate neutrophil and macrophage clearance in zebrafish and are differentially exploited by *Salmonella* Typhimurium

**DOI:** 10.1038/s41419-025-08291-8

**Published:** 2025-12-08

**Authors:** Juan M. Lozano-Gil, Annamaria Pedoto, Ana M. Conesa-Hernández, María Ocaña-Esparza, Victoriano Mulero, Sylwia D. Tyrkalska

**Affiliations:** 1 Departamento de Biología Celular e Histología, Facultad de Biología, Universidad de Murcia, Murcia, Spain; 2 Instituto Murciano de Investigación Biosanitaria Pascual Parrilla (IMIB-PP), Murcia, Spain; 3 Centro de Investigación Biomédica en Red de Enfermedades Raras (CIBERER), Instituto de Salud Carlos III, Madrid, Spain

**Keywords:** Apoptosis, Necroptosis, Phagocytes, Neutrophils

## Abstract

Programmed cell death (PCD) is essential for immune cell homeostasis and host defense, yet its role in neutrophil and macrophage elimination during bacterial infections remains poorly understood. Using the zebrafish model, which offers unique in vivo imaging and genetic manipulation advantages, we dissected the contribution of pyroptosis, apoptosis, and necroptosis to the regulation of neutrophil and macrophage fate during homeostasis and infection with *Salmonella enterica* serovar Typhimurium (ST). Under basal conditions, all three PCD pathways cooperated to control immune cell turnover. Upon infection, zebrafish larvae mounted a type III secretion system (T3SS)-independent emergency myelopoietic response that increased myeloid cell numbers. However, the pathogen rapidly counteracted this response by promoting neutrophil death through Nlrp3-mediated pyroptosis and Caspase-3-dependent apoptosis, and macrophage killing via Ripk1-dependent necroptosis—both driven by its T3SS. While blocking pyroptosis prevented neutrophil loss, it also increased host susceptibility due to impaired bacterial clearance, whereas inhibition of apoptosis or necroptosis enhanced resistance, as these pathways are dispensable for controlling infection. These findings demonstrate how ST exploits distinct PDC mechanisms to evade innate immunity and underscore their differential potential as therapeutic targets in intracellular bacterial infections.

## Introduction

Two primary mechanisms regulate the number of innate immune cells under both homeostatic and infection conditions: hematopoiesis and cell death. While hematopoiesis, including emergency hematopoiesis, has been extensively studied in recent years, the role of cell death in immune cell regulation has received comparatively less attention. Cell death is a fundamental biological process in which a cell ceases to function, playing a critical role in maintaining tissue homeostasis and embryonic development [1]. This process may occur naturally, as aging cells are replaced by new ones, or as a consequence of pathological conditions, injury, or organismal death [2]. Based on morphological and biochemical characteristics, cell death is classified into two main types: accidental cell death (ACD) and programmed cell death (PCD) [3, 4]. ACD occurs due to acute cellular injury, while PCD is a regulated process that plays essential roles in homeostasis, immunity, and disease.

A specialized form of lytic PCD, known as pyroptosis, is triggered by intracellular pathogen infections and is initiated through the formation of a large multiprotein complex called the inflammasome [5]. The canonical pyroptotic pathway is mediated by Caspase-1 (CASP1), which promotes the release of proinflammatory cytokines and intracellular contents [6]. Gasdermin-D (GSDMD), a member of the gasdermin protein family, plays a pivotal role in pyroptotic cell death as a key substrate of inflammatory caspases and, therefore, has emerged as a crucial mediator of host defense by responding to danger signals. Upon cleavage, the N-terminal fragment of GSDMD inserts into the membrane, forming pores that facilitate the efflux of small intracellular molecules. This process is further amplified by Ninjurin-1, which mediates plasma membrane rupture, allowing the release of larger intracellular molecules [7–12]. Notably, the cell swelling and lysis prevent intracellular pathogen replication and facilitate the release of proinflammatory cytokines, which recruit and activate immune cells at the site of infection or injury [13]. Remarkably, although various immune cells contribute to inflammatory responses, macrophages and neutrophils are the primary immune cells harboring active inflammasomes during infections and tissue injury [14].

In contrast to pyroptosis, apoptosis is a non-inflammatory PCD event [15]. It is a tightly regulated process that is initiated in response to cellular stress stimuli and, once triggered, proceeds irreversibly. Currently, two main pathways are known to activate apoptosis: the intrinsic pathway, which is dependent on the release of proteins from the mitochondrial intermembrane space, and the extrinsic pathway, which is initiated by extracellular ligands binding to cell-surface death receptors, leading to the formation of the death-inducing signaling complex (DISC) [16]. Both pathways converge on the activation of a cascade of caspases, distinct from CASP1 (i.e., CASP3 and CASP9), which orchestrate a series of molecular events culminating in controlled cell elimination [17, 18]. This cascade also facilitates the irreversible breakdown of the nuclear lamina, a key barrier to DNase activation, thereby enabling DNA fragmentation. Apoptotic cells exhibit characteristic morphological changes, including membrane blebbing, cell shrinkage, chromatin condensation, nuclear fragmentation, DNA degradation, and mRNA decay [19]. Importantly, the apoptotic cells dye silently, being rapidly engulfed by phagocytes before intracellular contents are released, thereby avoiding inflammation [20].

Finally, necroptosis has similar morphological features to apoptosis and necrosis. The discovery of necroptosis demonstrated that necrotic cell death can occur in a regulated manner, typically in a caspase-independent fashion and often when apoptosis is inhibited. The key mediators of necroptosis include receptor-interacting serine/threonine kinases 1 and 3 and their downstream effector, mixed lineage kinase domain-like protein [21]. Necroptosis functions as a host defense mechanism against infections, particularly viral pathogens. Morphologically, necroptotic cells exhibit organelle and cellular swelling, plasma membrane rupture, and a lack of nuclear fragmentation, ultimately resulting in cell lysis [22]. Unlike apoptosis, necroptosis leads to the uncontrolled release of intracellular contents, which can provoke a potent inflammatory response in neighboring cells [23].

In this study, we aimed to investigate the impact of different PCD pathways in neutrophils and macrophages on the progression of inflammation, with a particular focus on inflammasome-dependent cell death, using in vivo zebrafish-*Salmonella enterica* serovar Typhimurium (ST) infection models [24, 25]. This model offers the unique advantage of a vertebrate model for studying the role of PDC in innate immune responses through real-time in vivo imaging, while its genetic tractability and rapid development facilitate precise manipulation of immune pathways [26]. Our results revealed the complexity of cell death mechanisms of neutrophils and macrophages, and their hijacking by ST.

## Results

### *Salmonella* Typhimurium kills zebrafish neutrophils, but not macrophages, by Nlrp3/Gsdme-mediated pyroptosis

**Fig. 1:**
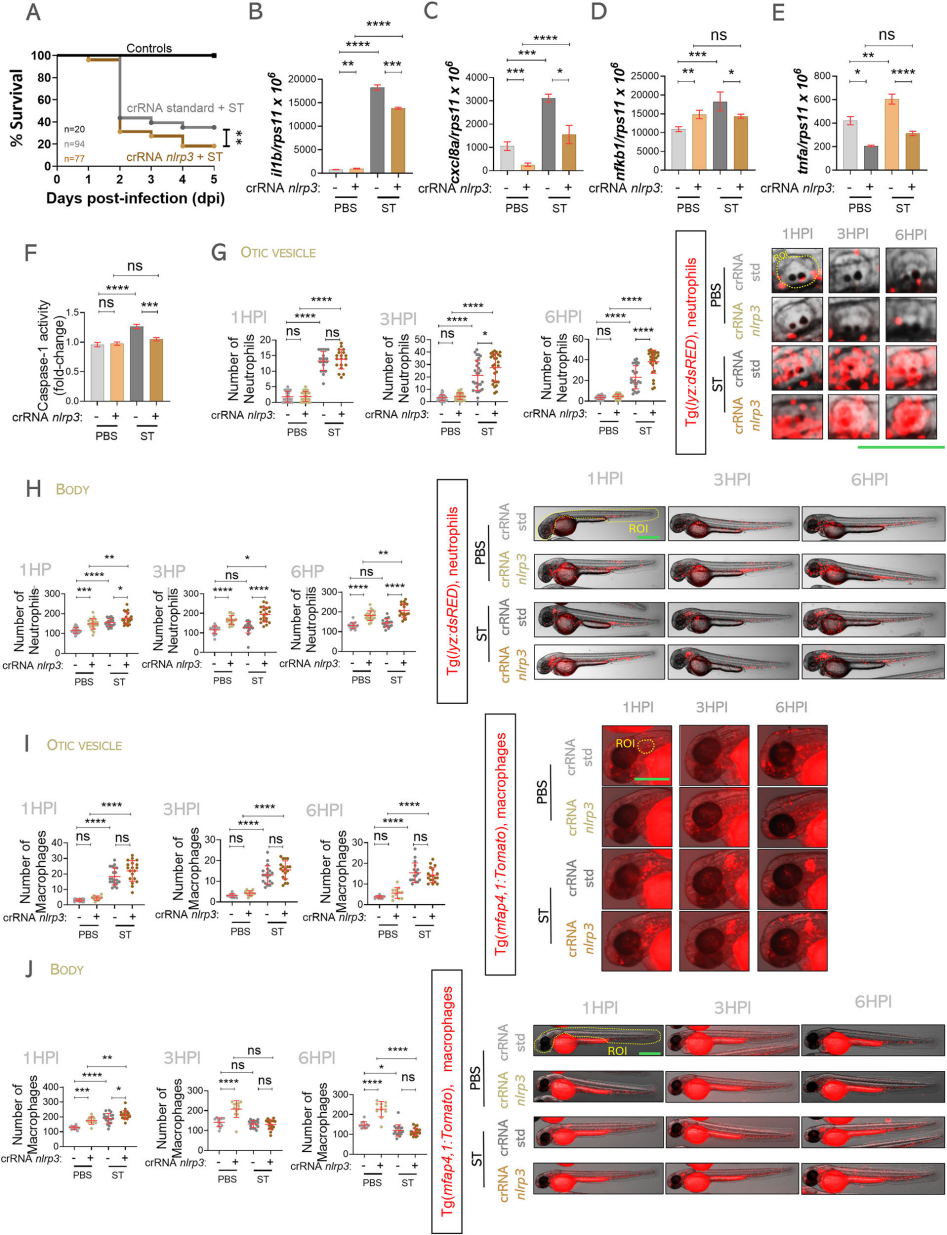
Nlrp3 inhibition blocks pyroptotic death of zebrafish neutrophils during *S.* Typhimurium infection. One-cell stage embryos were injected with standard or nlrp3 gRNAs/Cas9 complexes, then 2 dpf embryos were infected in yolk sac (**A**, **B**, **C**, **D**, **E**, **F**) or in the otic vesicle with wild-type strain of ST (**G**, **H**, **I**, **J**), controls were injected with PBS. **A** Susceptibility to ST was evaluated until 5 dpi. **B**–**E** The transcript levels of the indicated genes were analyzed at 24 hpi by RT-qPCR. **F** Caspase-1 activity was determined at 24 hpi using a fluorogenic substrate. **G**–**J** Neutrophil and macrophage recruitment to otic vesicle or total count was followed up at 1, 3 and 6 hpi. Each dot represents one individual, and the means ± SEM for each group is also shown. *P* values were calculated using one-way analysis of variance (ANOVA) and Tukey multiple range test. ns not significant; *P ≤ 0.05, **P ≤ 0.01, ***P ≤ 0.001, and ****P ≤ 0.0001. The regions of interest (ROI) used for quantification in all experiments are indicated in the representative images. Bars: 500 µm.

The zebrafish–*S*. Typhimurium infection model is widely used to study inflammasome activation and function, as well as its role in bacterial clearance. Two primary inflammasomes implicated in the host response to *S*. Typhimurium infection are NLRP4 and NLRP3. NLRP4 is predominantly involved in the early response, recognizing bacterial type 3 secretion system (T3SS) and flagellin, whereas NLRP3 plays a crucial role at later stages following bacterial replication [27–29]. In this study, we focused on the role of the Nlrp3 inflammasome during ST infection, since this inflammasome has been found to regulate neutrophil and macrophage numbers in zebrafish larvae under homeostasis and sterile inflammation [30, 31]. Knockdown of Nlrp3 by CRISPR-Cas9 (edition efficiency of about 75%) [30] resulted in higher susceptibility to ST infection (Fig. 1A), suggesting that Nlrp3 is crucial for host survival and bacterial clearance. Furthermore, Nlrp3 knockdown resulted in reduced expression of pro-inflammatory genes, including *il1b, cxcl8a*, *nfkb1* and *tnfa* at 24 h post-infection (hpi) (Fig. 1B–E). As expected, the activity of caspase a (Caspa), the functional homolog of mammalian CASP1 [32], was found to decrease in Nlrp3-deficient larvae (Fig. 1F). Using zebrafish reporter lines with fluorescently labeled neutrophils [*Tg(lyz:dsRED)*] and macrophages [*Tg(mfap4:Tomato)*], we analyzed the recruitment and total numbers of these immune cells in the otic vesicle following infection. Nlrp3 deficiency resulted in enhanced neutrophil recruitment at 3 and 6 hpi (Fig. 1G), likely reflecting an overall increase in neutrophil abundance in Nlrp3-deficient larvae under both homeostatic and infectious conditions (Fig. 1H). Interestingly, while infection induced a transient rise in neutrophil numbers at 1 hpi in both wild-type and Nlrp3-deficient larvae, only wild-type larvae showed a marked reduction in neutrophil counts at 3 and 6 hpi (Fig. 1H). This suggests that ST exploits Nlrp3 to selectively eliminate neutrophils during infection. In contrast, macrophage recruitment was not affected by Nlrp3 status (Fig. 1I). Although Nlrp3 inhibition led to increased macrophage numbers in homeostasis and at early infection timepoints, it did not prevent ST-induced macrophage loss at 3 and 6 hpi (Fig. 1J), indicating that Nlrp3 is involved in regulating macrophage homeostasis but not their killing during infection.

**Fig. 2:**
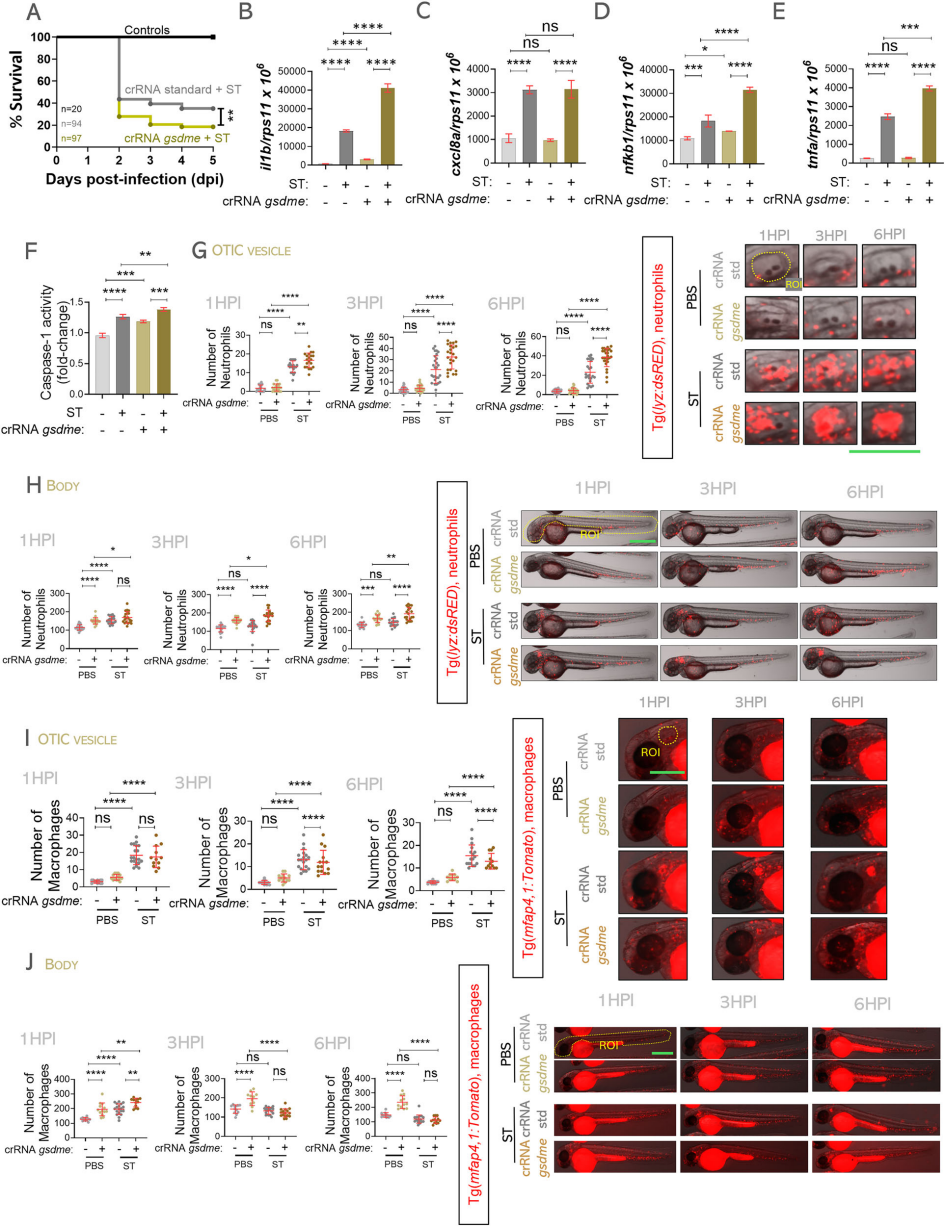
Gsdme - dependent pyroptosis selectively eliminates zebrafish neutrophils during *S*. Typhimurium infection. One-cell stage embryos were injected with standard or gsdmea and gsdmeb gRNAs/Cas9 complexes, then 2 dpf embryos were infected in yolk sac (**A**, **B**, **C**, **D**, **E**, **F**) or in the otic vesicle with wild-type strain of ST (**G**, **H**, **I**, **J**), controls were injected with PBS. **A** Susceptibility to ST was evaluated until 5 dpi. **B**–**E** The transcript levels of the indicated genes were analyzed at 24 hpi by RT-qPCR. **F** Caspase-1 activity was determined at 24 hpi using a fluorogenic substrate. **G**–**J** Neutrophil and macrophage recruitment to otic vesicle or total count was followed up at 1, 3 and 6 hpi. Each dot represents one individual, and the means ± SEM for each group is also shown. *P* values were calculated using one-way analysis of variance (ANOVA) and Tukey multiple range test. ns not significant; *P ≤ 0.05, **P ≤ 0.01, ***P ≤ 0.001, and ****P ≤ 0.0001. The regions of interest (ROI) used for quantification in all experiments are indicated in the representative images. Bars: 500 µm.

Gasdermin D is a key executor of pyroptosis in mammals, with zebrafish homologs Gasdermin Ea and Eb (Gsdmea and Gsdmeb) [33]. To determine whether Gsdme operates downstream of Nlrp3 in zebrafish, we knocked down Gsdmea and Gsdmeb using specific gRNAs (~60% editing efficiency) [30]. Gsdme deficiency increased susceptibility to ST infection (Fig. 2A), like Nlrp3 deficiency. However, in contrast to Nlrp3 knockdown, Gsdme depletion led to elevated levels of inflammatory markers (*il1b*, *cxcl8a*, *nfkb1*, *tnfa*) and enhanced CASP1 activity at 24 hpi (Fig. 2B–F), likely due to the loss of Gsdme-mediated inhibition of Caspa [31]. Regarding immune cell dynamics, we observed the same pattern as with Nlrp3 deficiency: increased neutrophil recruitment and total numbers under both homeostasis and infection, with selective neutrophil elimination by ST in a Gsdme-dependent manner (Fig. 2G, H). Macrophage recruitment was slightly impaired in Gsdme-deficient larvae at 3 and 6 hpi, and although Gsdme deficiency increased macrophage counts in basal and early infection stages, bacterial killing reduced macrophage numbers at later timepoints (Fig. 2I–J). Thus, the Nlrp3/Gsdme inflammasome axis governs homeostatic turnover of both neutrophils and macrophages, but during infection, ST exploits this pathway specifically to kill neutrophils.

To confirm that the reduction in neutrophil numbers during ST infection results from inflammasome-dependent cell death, we performed TUNEL staining in Nlrp3-deficient larvae. Nlrp3 knockdown led to a marked decrease in TUNEL-positive cells in the head at the site of the infection at 4 hpi following localized infection, indicating that Nlrp3 is required for infection-induced cell death (Fig. S1A). To determine whether neutrophils were specifically affected, we combined TUNEL staining with whole-mount immunohistochemistry for neutrophil detection using an antibody to zebrafish Mpx. At 2 hpi, no significant differences in TUNEL^+^ neutrophils were observed between groups in either the head or whole larvae, regardless of infection status. However, at 4 hpi, Nlrp3-deficient larvae exhibited a significant reduction in TUNEL^+^ neutrophils compared to controls, both in the head and across the body, but only under infected conditions (Fig. S1B). These findings confirm that ST induces Nlrp3-dependent neutrophil death during infection, supporting the idea that the decline in neutrophil numbers is primarily due to pyroptosis.

### Zebrafish neutrophil and macrophage cell death depends on Type 3 Secretion System of *Salmonella* Typhimurium

**Fig. 3:**
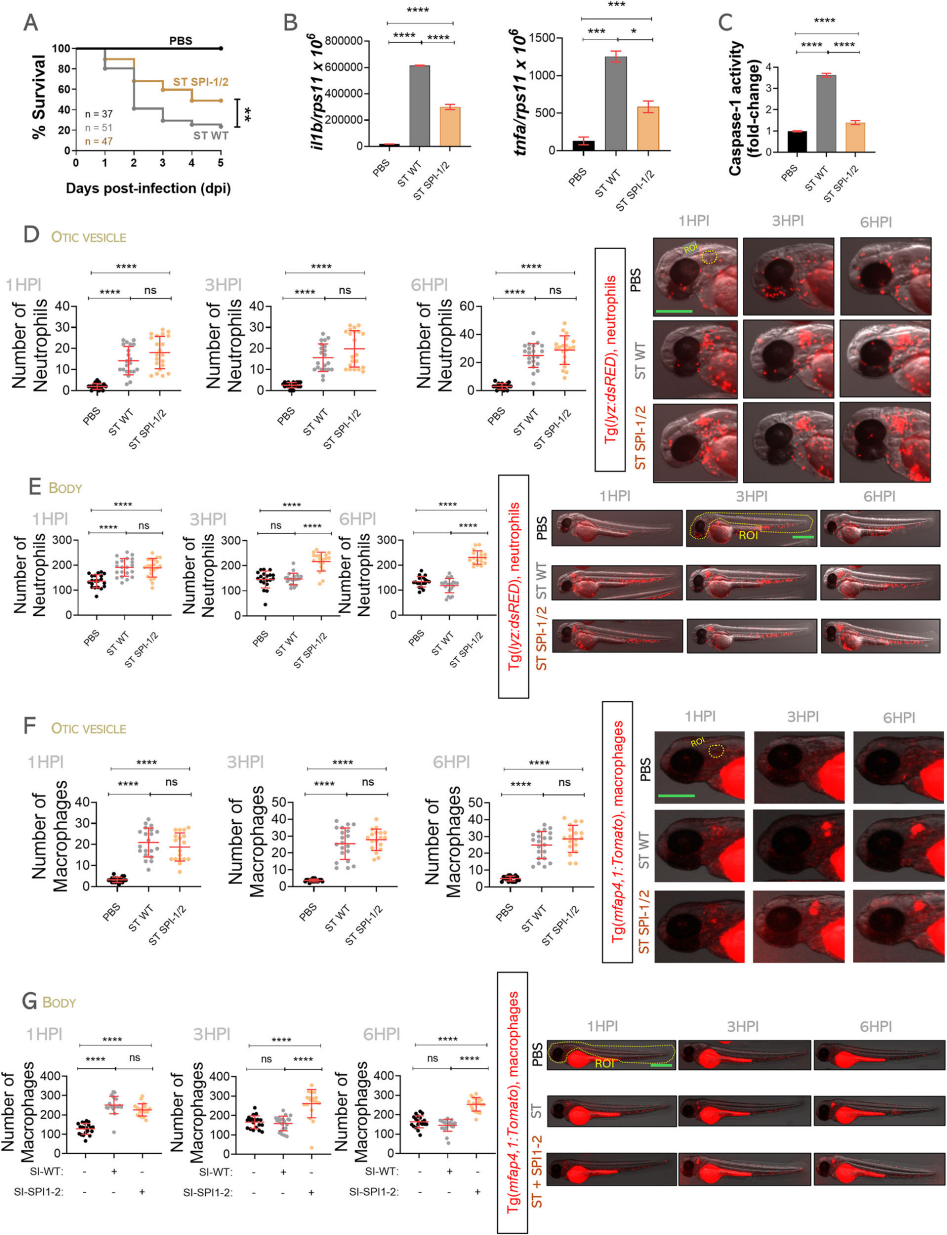
*S.* Typhimurium triggers neutrophil and macrophage cell death in zebrafish through its Type III Secretion System. Wild-type 2 dpf embryos were infected in yolk sac (**A**, **B**, **C**) or in the otic vesicle with wild-type (WT) or the isogenic SPI-1/2 mutant (SPI-1/2) strain of ST (**D**, **E**, **F**), controls were injected with PBS. **A** Susceptibility to ST was evaluated until 5 dpi. **B** The transcript levels of the indicated genes were analyzed at 24 hpi by RT-qPCR. **C** Caspase-1 activity was determined at 24 hpi using a fluorogenic substrate. **D**–**G** Neutrophil and macrophage recruitment to otic vesicle or total count was followed up at 1, 3 and 6 hpi. Each dot represents one individual, and the means ± SEM for each group is also shown. *P* values were calculated using one-way analysis of variance (ANOVA) and Tukey multiple range test. ns not significant; *P ≤ 0.05, **P ≤ 0.01, ***P ≤ 0.001, and ****P ≤ 0.0001. The regions of interest (ROI) used for quantification in all experiments are indicated in the representative images. Bars: 500 µm.

The virulence of ST depends on its T3SS, which is encoded by *Salmonella* pathogenicity island 1 and 2 (SPI-1 and SPI-2) [34, 35]. SPI-1 is responsible for host cell invasion and immune response modulation, whereas SPI-2 is essential for intracellular survival and replication [34, 35]. To investigate whether the absence of the T3SS affects host cell death, we infected zebrafish larvae with either wild-type *S*. Typhimurium or its isogenic SPI-1/2 mutant. Larvae infected with the SPI-1/2 mutant showed significantly higher survival rates than those infected with the wild-type strain (Fig. 3A), consistent with previous findings [24]. Moreover, the mutant induced markedly lower levels of inflammation and CASP1 activity at 24 hpi (Fig. 3B–C), supporting the notion that the T3SS is a key driver of Nlrp3 inflammasome activation and the associated inflammatory response. Furthermore, recruitment of neutrophils and macrophages to the infection site was not affected by the absence of the T3SS (Fig. 3D–F). However, only wild-type ST induced a significant reduction in total neutrophil and macrophage numbers at 3 and 6 hpi, consistent with T3SS-dependent cell death (Fig. 3E–G). In contrast, the SPI-1/2 mutant preserved immune cell viability throughout infection, underscoring the essential role of the T3SS in mediating inflammasome-driven killing of both neutrophils and macrophages.

### *Salmonella* Typhimurium also kills zebrafish neutrophils, but not macrophages, by apoptosis

**Fig. 4:**
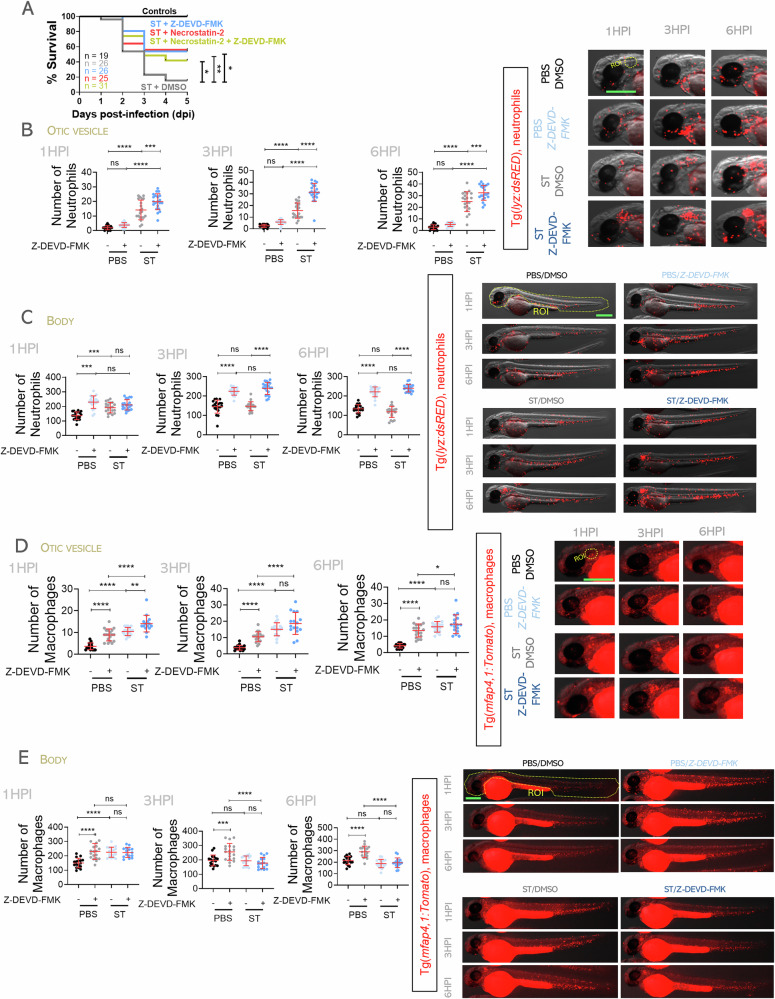
*S.* Typhimurium induces apoptotic death in zebrafish neutrophils while sparing macrophages. Wild-type 2 dpf embryos were infected in yolk sac (**A**) or in the otic vesicle with wild-type (WT) strain of ST (**B**-**E**), controls were injected with PBS. From 1 h before ST injection, embryos were treated with the inhibitor of Caspase-3 (Z-DEVD-FMK) or and/or the necroptosis inhibitor (necrostatin-2) or DMSO. **A** Susceptibility to ST was evaluated until 5 dpi. **B**–**E** Neutrophil and macrophage recruitment to otic vesicle or total count was followed up at 1, 3 and 6 hpi. Each dot represents one individual, and the means ± SEM for each group is also shown. *P* values were calculated using one-way analysis of variance (ANOVA) and Tukey multiple range test. ns not significant; *P ≤ 0.05, **P ≤ 0.01, ***P ≤ 0.001, and ****P ≤ 0.0001. The regions of interest (ROI) used for quantification in all experiments are indicated in the representative images. Bars: 500 µm.

To assess the contribution of apoptosis to immune cell death during ST infection, we treated larvae with the caspase-3 (Casp3) inhibitor Z-DEVD-FMK. Inhibition of Casp3 enhanced larval survival (Fig. 4A), confirming previous results [36], and increased neutrophil recruitment and total numbers at 3 and 6 hpi (Fig. 4B, C), indicating that neutrophils undergo both homeostatic and infection-induced apoptosis. In contrast, macrophage recruitment was unaffected (Fig. 4D), and total macrophage numbers remained reduced at 3 and 6 hpi despite Casp3 inhibition (Fig. 4E), suggesting that ST kills macrophages through a Casp3-independent mechanism. Furthermore, genetic inhibition of Casp3a (edition efficiency of about 34%), which is the only one paralog expressed by neutrophils according to DanioCell [37], further confirmed the killing of neutrophils by ST through apoptosis (Fig. S2A, B). Moreover, genetic inhibition of caspase-activated DNase (Cad), an endonuclease that mediates DNA fragmentation during apoptosis, through overexpression of its endogenous inhibitor Icad [38], together with pharmacological inhibition of Caspase-9 (Ac-LEHD-CMK), a key enzyme of the intrinsic apoptotic pathway, confirmed the involvement of neutrophil apoptosis during infection, yielding the same outcome as Casp3 inhibition (Fig. S3A–D). Together, these data indicate that apoptosis contributes to homeostatic clearance of both neutrophils and macrophages, but during infection, ST specifically targets neutrophils via combined pyroptotic and apoptotic mechanisms, implying the involvement of an alternative PDC pathway for macrophage elimination.

### *Salmonella* Typhimurium kills zebrafish neutrophils and macrophages by necroptosis

**Fig. 5:**
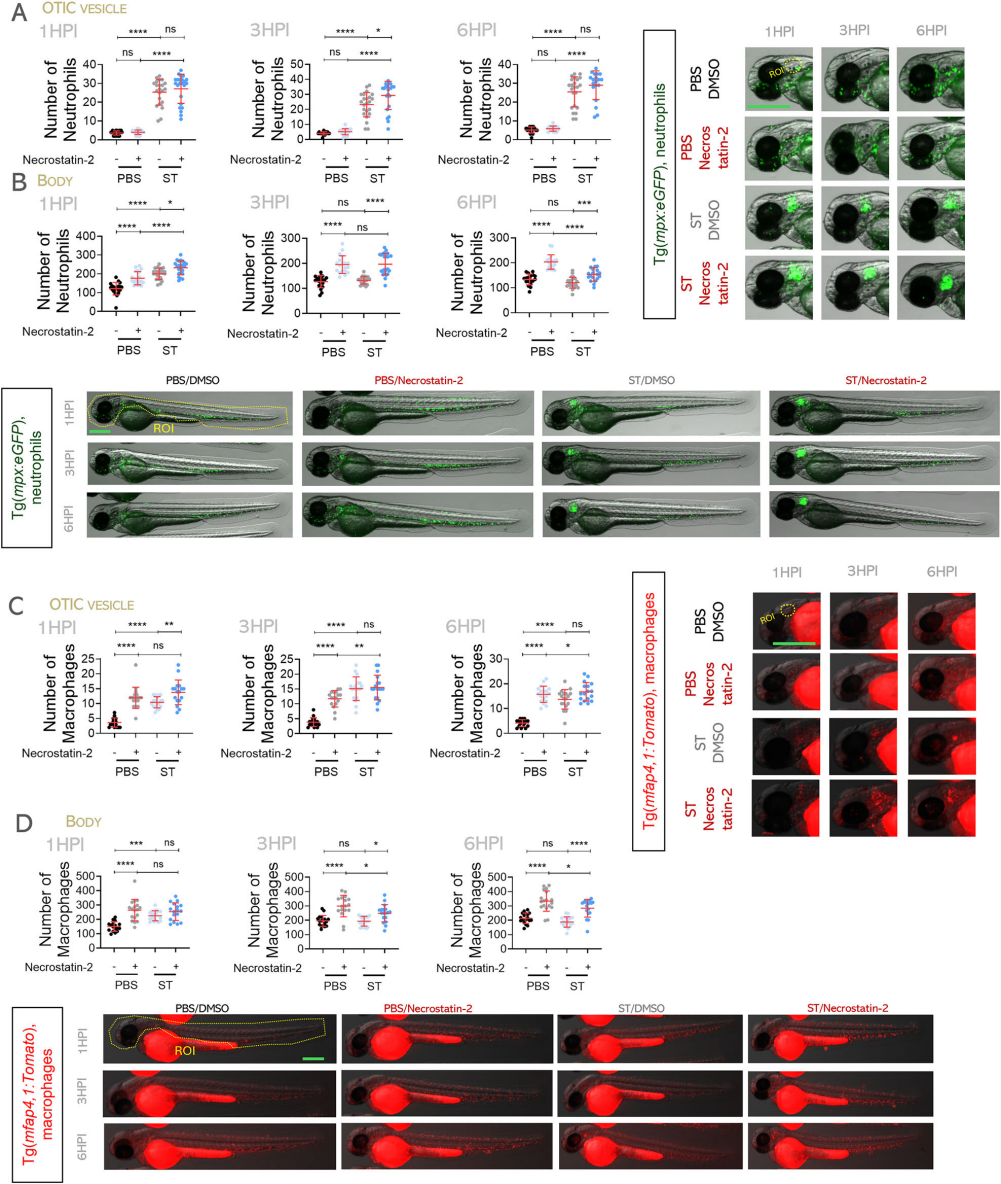
*S.* Typhimurium induces necroptotic death of zebrafish neutrophils and macrophages. Wild-type 2 dpf embryos were infected the otic vesicle with wild-type (WT) strain of ST (**A**-**D**), controls were injected with PBS. From 1 h before ST injection, embryos were treated with the necroptosis inhibitor (necrostatin-2) or DMSO. **A**–**D** Neutrophil and macrophage recruitment to otic vesicle or total count was followed up at 1, 3 and 6 hpi. Each dot represents one individual, and the means ± SEM for each group is also shown. *P* values were calculated using one-way analysis of variance (ANOVA) and Tukey multiple range test. ns, not significant; *P ≤ 0.05, **P ≤ 0.01, ***P ≤ 0.001, and ****P ≤ 0.0001. The regions of interest (ROI) used for quantification in all experiments are indicated in the representative images. Bars: 500 µm.

To determine whether necroptosis contributes to macrophage elimination during ST infection, we inhibited Ripk1 using necrostatin-2. Treated larvae showed increased survival, comparable to Casp3 inhibition, but no further protection was observed when both pathways were inhibited simultaneously (Fig. 4A). Neutrophil recruitment was largely unaffected (Fig. 5A), but total neutrophil numbers increased in homeostasis and were partially preserved upon infection (Fig. 5B), suggesting a role for necroptosis in their basal turnover and delayed infection-induced death. Macrophage recruitment remained mostly unchanged (Fig. 5C), but their total numbers increased under both basal and infection conditions following Ripk1 inhibition, indicating that necroptosis is critical for macrophage elimination by ST (Fig. 5D). The relevance of necroptosis in the elimination of macrophages by ST was further confirmed by genetic inactivation of Ripk1 (edition efficiency of about 40%) (Fig. S2C, D). Overall, these findings reveal that apoptosis, pyroptosis, and necroptosis all regulate homeostatic turnover of neutrophils and macrophages in zebrafish larvae. During infection ST exploits pyroptosis and apoptosis to kill neutrophils and necroptosis to eliminate macrophages.

## Discussion

**Fig. 6:**
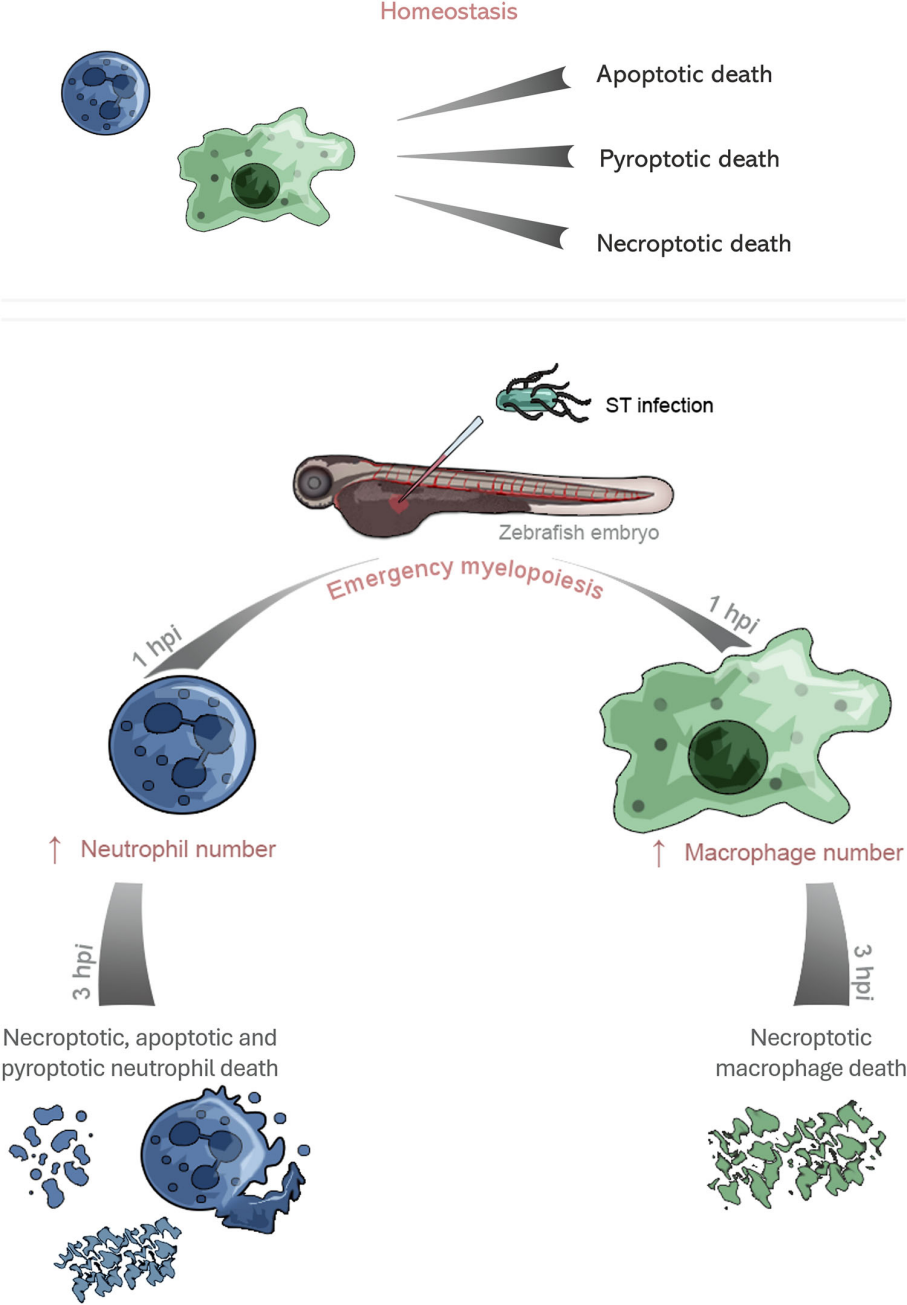
Schematic representation of neutrophil and macrophage cell death under homeostatic and *Salmonella* Typhimurium (ST) infection conditions. Under homeostatic conditions, neutrophils and macrophages undergo turnover through regulated cell death mechanisms, including apoptosis, pyroptosis, and necroptosis. Initially, upon *S*. Typhimurium infection, there is a rapid increase in neutrophil and macrophage numbers due to emergency hematopoiesis. However, as early as 3 h post-infection, neutrophils begin to undergo all three types of cell death: apoptosis, pyroptosis, and necroptosis, while macrophages predominantly die via necroptosis. This shift highlights the dynamic and differential regulation of phagocyte cell death in response to bacterial infection.

Cell death is a critical process that occurs during development and throughout an individual’s life. It involves the complete and irreversible cessation of biochemical activities in a living cell. Cell death is also a common outcome during infections, with the mode of death influenced by factors such as pathogen type, pathogen load, and the infection site [39]. Under physiological homeostasis, human and mouse neutrophils exhibit a lifespan of approximately 8–20 h, after which they undergo constitutive, spontaneous apoptosis [40]. The clearance of apoptotic neutrophils in vivo is mediated by tissue-resident macrophages in the spleen and bone marrow, as well as Kupffer cells in the liver, the whole process being conserved between the species [41–43]. In addition, recent studies have demonstrated that GSDMD-mediated pyroptosis also contributes to constitutive neutrophil death under homeostatic conditions in mice in vivo. [44, 45]. In contrast to neutrophils, macrophages are generally regarded as long-lived cells, with lifespans that can extend for several months under steady-state conditions. While their homeostatic cell death has not been extensively investigated in vivo, their death mechanisms have been more thoroughly characterized under inflammatory conditions. In our zebrafish model, we analyzed cell death mechanisms in homeostatic conditions in neutrophils and macrophages in vivo, finding that all three: pyroptosis, apoptosis, and necroptosis operate simultaneously to regulate the clearance of these innate immune cells. The zebrafish model provides a unique advantage for studying cell death in vivo under homeostatic conditions, as it allows direct visualization and differentiation of apoptosis, pyroptosis, and necroptosis in both neutrophils and macrophages (Fig. 6). This contrasts with most murine models, where such resolution of simultaneous and cell-type-specific death pathways in steady state is limited. The zebrafish system thus offers a powerful platform to dissect the physiological roles and regulation of distinct cell death mechanisms in innate immune cells in vivo.

The interplay between host and pathogen is a dynamic and reciprocal process that drives the evolution of host immune defenses. In the context of intracellular bacterial infections, pyroptosis serves as a key defense strategy to disrupt intracellular replication niches and amplify immune responses. It is well established that both neutrophils and macrophages can undergo pyroptosis in response to bacterial pathogens in vivo in mouse models, including ST infections [46–49]. However, using the zebrafish ST infection model, we found that ST kills neutrophils by Nlrp3-Gsdme-dependent pyroptosis, whereas this form of cell death is not involved in macrophage killing. As mentioned, neutrophil pyroptosis occurs in both zebrafish and mouse models upon bacterial infections, underscoring the evolutionary conservation and biological relevance of this cell death pathway. In contrast, the inability of ST to kill macrophages by pyroptosis in zebrafish highlights species-specific differences, guiding researchers to use complementary models when studying macrophage responses. Supporting our findings, we have recently reported that blocking the inflammasome specifically in neutrophils and macrophages by overexpressing a dominant-negative form of Asc repressed pyroptotic cell death of both cell types in noninfected larvae but only of neutrophils after ST infection [31]. In addition, overactivation of the neutrophil and macrophage inflammasome by overexpression of wild-type Asc resulted in cell-autonomous, Gsdme-mediated neutrophil and macrophage pyroptotic cell death under basal and infection conditions [31]. Therefore, although ST is unable to kill zebrafish macrophages via pyroptosis, hyperactivation of the inflammasome by overexpression of Asc led to Gsdme-dependent macrophage pyroptotic cell death (Fig. 6).

If a cell is unable to fully undergo pyroptosis, alternative pathways will trigger apoptosis as a backup mechanism. This phenomenon has been inadequately explored in vivo in relation to neutrophils and macrophages. Limited in vivo studies have demonstrated that murine macrophages can undergo apoptosis during ST infections [50, 51]. Similarly, in the context of ST infection, necroptosis has so far been observed exclusively in macrophages in vivo in mouse models, with no available data currently supporting its occurrence in neutrophils. [52, 53]. Mammalian neutrophils can undergo necroptosis; however, they need different stimuli, such as phagocytosis of *Staphylococcus aureus* [54]. In the in vivo zebrafish model, we showed that neutrophils undergo Casp3- and Caspase-9-dependent apoptosis during ST infection, indicating that the intrinsic apoptotic pathway is engaged, a process not observed in macrophages. In contrast, Ripk1-dependent necroptosis affected both neutrophils and macrophages. These findings highlight the zebrafish as a powerful in vivo system to study necroptosis across multiple immune cell types, offering a dynamic and transparent platform to investigate neutrophil and macrophage responses during infection. Overall, ST infection induces pyroptosis, apoptosis, and necroptosis in zebrafish neutrophils, whereas macrophages are predominantly killed via necroptosis (Fig. 6). The rescue of neutrophils upon inhibition of any single pathway suggests that all three are activated simultaneously, indicating that ST may trigger panoptosis in neutrophils. Further studies are needed to confirm this. The zebrafish-ST model offers a unique in vivo tool to explore the role of panoptosis in clearing intracellular bacterial infections—an area mostly studied in vitro using mouse macrophages.

The balance between blood cell production and cell death determines the final number of neutrophils and macrophages in circulation. Both processes are regulated by the inflammasome, a cytosolic multiprotein complex with the effector CASP1, which can activate myelopoiesis and pyroptotic cell death under homeostatic conditions and during infection [55–57]. Our study established on the one hand that the Nlrp3-Gsdme inflammasome plays a critical role in mediating neutrophil pyroptosis under both homeostatic and infectious conditions, while it is essential for macrophage pyroptosis specifically in homeostasis. On the other hand, while ST infection triggers emergency myelopoiesis, marked by an early increase in neutrophil and macrophage numbers, this process appears to be independent of the T3SS. Instead, it is likely driven by the inflammatory response itself, as similar hematopoietic changes are observed in sterile inflammation models such as silicosis and COVID-19–associated cytokine storm syndrome [30, 58]. However, the ST T3SS mediates the rapid killing of both neutrophils and macrophages, confirming previous studies in mice showing that both SPI-1 and SPI-2 are required for the killing of macrophages by ST [59, 60]. As it has been demonstrated that rapid cell death relies on SPI-1, while delayed cell death is SPI-2 dependent, those two being important for understanding ST pathogenesis [59, 61], it will be worthy to analyze the contribution to each SPI to the killing of zebrafish neutrophils and macrophages by ST.

In summary, we report here a unique animal model to study in vivo the impact of inflammasome in inflammation, cell death and infectious disease progression. It gives the possibility to study the local and systemic inflammation simultaneously in a whole organism in vivo, cell-restricted expression of genes of interest and manipulation of different cell deaths. Using this model, we uncovered a previously unappreciated role of neutrophil and macrophage cell death, where the combination therapy targeting PCD signaling pathways may be potentially beneficial in certain intracellular bacterial infections like ST, as it has already been suggested in other diseases like different types of cancers, neurological, metabolic, and autoimmune disorders [62–67]. This strategy could represent a valuable therapeutic alternative for managing infections by intracellular bacteria that no longer respond to conventional antibiotics, addressing a critical and escalating public health concern.

## Materials and methods

### Animals

Zebrafish (*Danio rerio* H.) were obtained from the Zebrafish International Resource Center and mated, staged, raised and processed as described [68]. The lines *Tg(lyz:dsRED)*^*nz50*^ [69], *Tg(mpx:eGFP)*^*i114*^ [70], *Tg(mfap4:tomato)*^*xt12*^ [71] and casper (*mitfaw2/w2; mpv17a9/a9*) [72], were previously described. were generated in the house. The experiments performed comply with the Guidelines of the European Union Council (Directive 2010/63/EU) and the Spanish RD 53/2013. The experiments and procedures were performed approved by the Bioethical Committees of the University of Murcia (approval number #669/2020).

### CRISPR and RNA injection in zebrafish

crRNA for zebrafish *gsdmea*, *gsdmeb*, *nlrp3*, *ripk1, casp3a* and negative control crRNA (Catalog #1072544), and tracrRNA were purchased from IDT and resuspended in Nuclease-Free Duplex Buffer to 100 µM. 1 µl of each was mixed and incubated for 5 min at 95 °C for duplexing. After removing from the heat and cooling to room temperature, 1.43 µl of Nuclease-Free Duplex Buffer was added to the duplex, giving a final concentration of 1000 ng/µl. Finally, the injection mix was prepared by mixing 1 µl of duplex, 2.55 µl of Nuclease-Free Duplex Buffer, 0.25 µl Cas9 Nuclease V3 (IDT, #1081058) and 0.25 µl of phenol red, giving final concentrations of 250 ng/µl of gRNA duplex and 500 ng/µl of Cas9. The prepared mix was microinjected into the yolk of one- to eight-cell-stage embryos using a microinjector (Narishige) (0.5–1 nl per embryo). The same amounts of gRNA were used in all the experimental groups. The efficiency of gRNA was checked by amplifying the target sequence with a specific pair of primers (Table S1) and the TIDE webtool (https://tide.nki.nl/).

The coding sequence of zebrafish *icad* (NM_001002631) was synthesized by GeneScript. In vitro-transcribed RNA was obtained following manufacturer’s instructions (mMESSAGE mMACHINE kit, Ambion). RNA was mixed in microinjection buffer and microinjected into the yolk of one-cell-stage embryos using a microinjector (Narishige; 0.5–1 nl per embryo). The same amount of RNA was used for all the experimental groups. Overexpression of Icad has been previously validated by us to inhibit apoptosis in zebrafish embryos [38].

### Chemical treatments

For image acquisition and cell count experiments, 24-h postfertilization (hpf) embryos were manually dechorionated and treated until 48 hpf by bath immersion with 50 μM Z-DEVD-FMK (#HY-12466), 10 μM Necrostatin-2 (#HY-14622A) (all from MedchemExpress) and 50 μM Ac-LEHD-CMK (#218728, Sigma-Aldrich). In some experiments, 24 hpf embryos were treated with 0.3% N-Phenylthiourea (PTU, Sigma-Aldrich) to inhibit melanogenesis.

### Analysis of gene expression

Total RNA was extracted from whole larvae with TRIzol reagent (Invitrogen) following the manufacturer’s instructions and treated with DNase I, amplification grade (1 U/mg RNA: Invitrogen). SuperScript IV RNase H Reverse Transcriptase (Invitrogen) was used to synthesize first-strand cDNA with random primer from 1 µg of total RNA at 50 °C for 50 min. Real-time PCR was performed with an ABIPRISM 7500 instrument (Applied Biosystems) using SYBR Green PCR Core Reagents (Applied Biosystems). Reaction mixtures were incubated for 10 min at 95 °C, followed by 40 cycles of 15 s at 95 °C, 1 min at 60 °C, and finally 15 s at 95 °C, 1 min 60 °C, and 15 s at 95 °C. For each mRNA, gene expression was normalized to the ribosomal protein S11 gene (*rps11*) content in each sample, using the Pfaffl method [73]. The primers used are shown in Table S2. In all cases, each PCR was performed with triplicate samples and repeated at least with two independent samples. “*n*” for each experimental group: 10 larvae/treatment.

### Caspase-1 activity assays

The CASP1 activity was determined with the fluorometric substrate Z-YVAD 7-Amido-4-trifluoromethylcoumarin (Z-YVAD-AFC, CASP1 substrate VI, Calbiochem), as described previously [24, 55]. In brief, 25–35 whole larvae were lysed in hypotonic cell lysis buffer (25 mM 4-(2-hydroxyethyl) piperazine-1-ethanesulfonic acid, 5 mM ethylene glycol-bis(2-aminoethylether)-N,N,N´,N´-tetraacetic acid, 5 mM dithiothreitol, 1:20 protease inhibitor cocktail (Sigma-Aldrich), pH 7.5) on ice for 10 min. For each reaction, 100 µg protein was incubated for 90 min at room temperature with 50 mM YVAD-AFC and 50 µl of reaction buffer (0.2% 3-[(3-cholamidopropyl)dimethylammonio]-1-propanesulfonate (CHAPS), 0.2 M 4-(2-hydroxyethyl) piperazine-1-ethanesulfonic acid, 20% sucrose, 29 mM dithiothreitol, pH 7.5). After incubation, the fluorescence of the AFC released from the Z-YVAD-AFC substrate was measured with a FLUOstart spectofluorometer (BGM, LabTechnologies) at an excitation wavelength of 405 nm and an emission wavelength of 492 nm. A representative CASP1 activity graph out of three repeats is shown in figures. “*n*” for each experimental group: 30 larvae/treatment.

### Infection assays

For infection experiments, ST 12023 (WT) and the isogenic derivative double mutant for SPI-1/SPI-2 (prgH020::Tn5lacZY ssaV::aphT; kindly provided by Prof. D. Holden) were used. Overnight cultures in Luria-Bertani (LB) broth were diluted 1/5 in LB with 0.3 M NaCl, incubated at 37 °C until 1.5 optical density at 600 nm was reached, and finally diluted in sterile PBS. Larvae of 2 dpf were anaesthetized in embryo medium with 0.16 mg ml^−1^ tricaine and 10 bacteria (yolk sac) or 100 (otic vesicle) per larvae were microinjected. Larvae were allowed to recover in egg water at 28–29 °C and monitored for clinical signs of disease or mortality over 5 days. At least three independent experiments were performed, and the total number of larvae per treatment is given in the graphs.

### Imaging of zebrafish larvae

To study immune cell recruitment to the injection site, 2 dpf *Tg(mpx:eGFP)*, *Tg(lyz:dsRed)* or *Tg(mfap4:tomato)* larvae were anaesthetized in embryo medium with 0.16 mg/ml tricaine. Images of the otic vesicle and the whole-body areas were taken 1, 3, 6 or 24 h post-injection (hpi) using a Leica MZ16F fluorescence stereomicroscope. The number of neutrophils or macrophages was determined by counting visually, and the fluorescence intensity was obtained and analyzed with ImageJ (FIJI) software [74]. In all experiments, images were pooled from at least three independent experiments performed by two people and using blind samples with a total number of at least 15 larvae per treatment.

### TUNEL assay and immunohistochemistry staining

The number of apoptotic cells in 2 dpf larvae was assessed by TUNEL assay. The larvae were fixed with 4% paraformaldehyde for 2 h and dehydrated with methanol at –20 °C. After gradual rehydration, the embryos were treated with 100% acetone at –20 °C for 10 min and rinsed three times for 10 min with PBT (PBS with 0.1% Tween-20). Embryos were permeabilized with a solution containing 0.1% Triton X-100 and 0.1% sodium citrate in PBS for 30 min at room temperature, rinsed 5 times with PBST for 5 min each, and blocked in 10% calf serum, 1% DMSO, 0.1% Tween-20 for 1 h to initiate whole body immunohistochemistry staining (WIHC).

To label neutrophils, larvae were incubated with anti-Mpx antibody (Genetex #GTX128379, 1:150) overnight at 4 °C, rinsed 5 times for 5 min with PBST, incubated with a secondary antibody (Invitrogen #A21207, 1:250) for 1 h and finally, rinsed three times for 5 min with PBST and stored in 70% glycerol in PBS until image acquisition. Images were acquired in a Leica MZ16F fluorescence stereo microscope. In all experiments, images were pooled from at least two independent experiments with a total number of at least 5 larvae per treatment.

### Statistical analysis

Statistical analysis was performed using Prism 8.0 (GraphPad Software, CA, USA). No calculation was performed to predetermine sample size; experiments were repeated three times to ensure robustness. Embryos were randomly allocated to each experimental condition and analyzed in blind samples. No data were excluded from the analysis. Data are shown as mean ± s.e.m. and were analyzed by analysis of variance and a Tukey multiple range test to determine differences between groups. The differences between the two samples were analyzed by the two-sided Student’s *t* test. The data met the normal distribution assumption when required and showed similar variances. A log-rank test was used to calculate the statistical differences in the survival of the different experimental groups. A *p* < 0.05 was considered statistically significant.

## Supplementary information

Figure S1

Figure S2

Figure S3

Supplementary Figures Legends

Table S1

Table S2

## Data Availability

All data generated or analyzed during this study are publicly available in the BioStudies database under accession number S-BIAD2361. The dataset can be accessed at https://www.ebi.ac.uk/biostudies/bioimages/studies/S-BIAD2361?key=4eab5ecd-02fa-4e46-876f-ceb9f964352a and is referenced by DOI 10.6019/S-BIAD2361.
