# Supplementary figures and images for: Programmed cell death pathways coordinate neutrophil and macrophage clearance in zebrafish and are differentially exploited by Salmonella Typhimurium
Source: Cell Death Dis. 2025 Dec 8;17(1):86. doi: 10.1038/s41419-025-08291-8 (PMC12830592; doi:10.1038/s41419-025-08291-8)

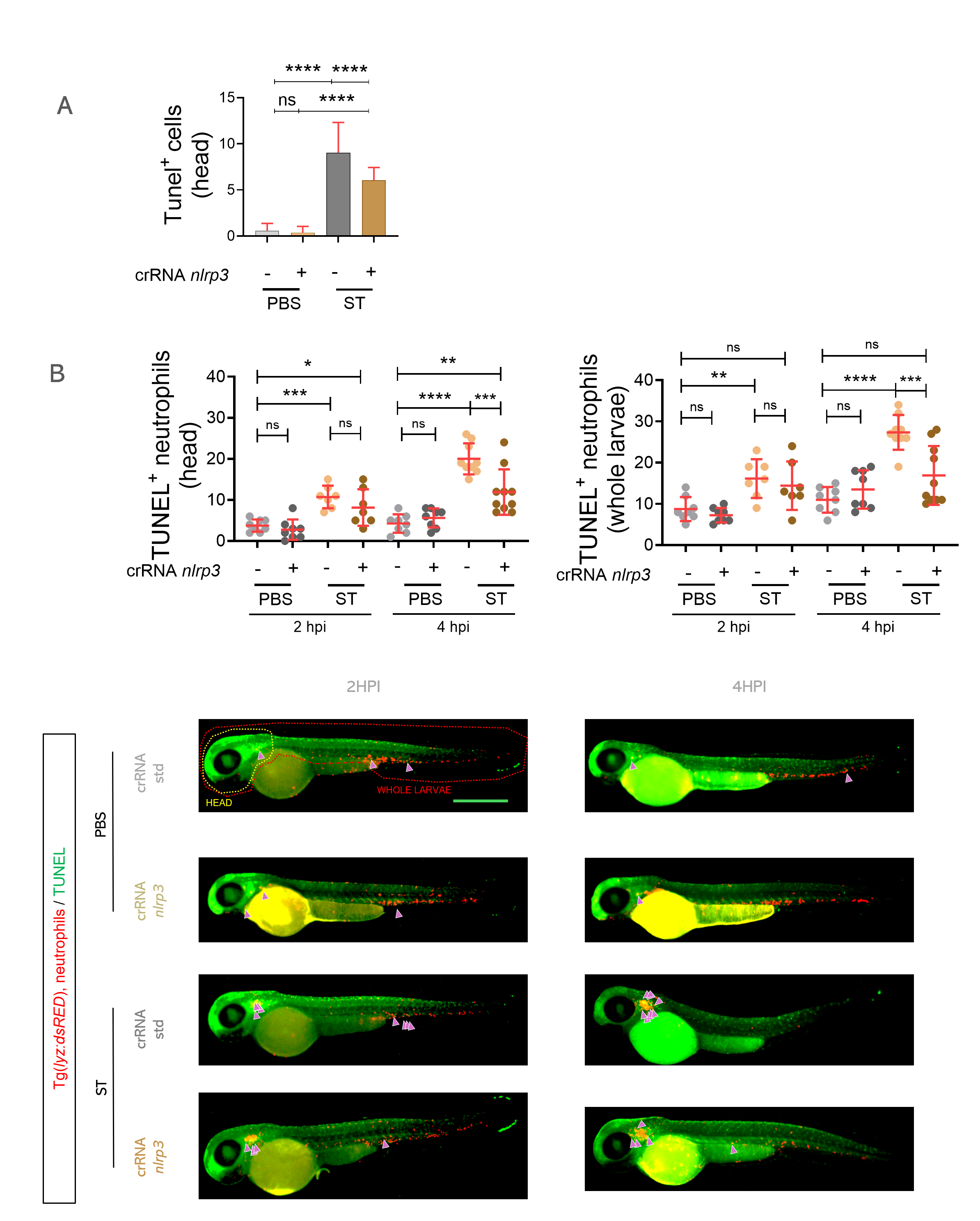

Supplement: Supplementary file 1 — Figure S1 [file 41419_2025_8291_MOESM1_ESM.tif]

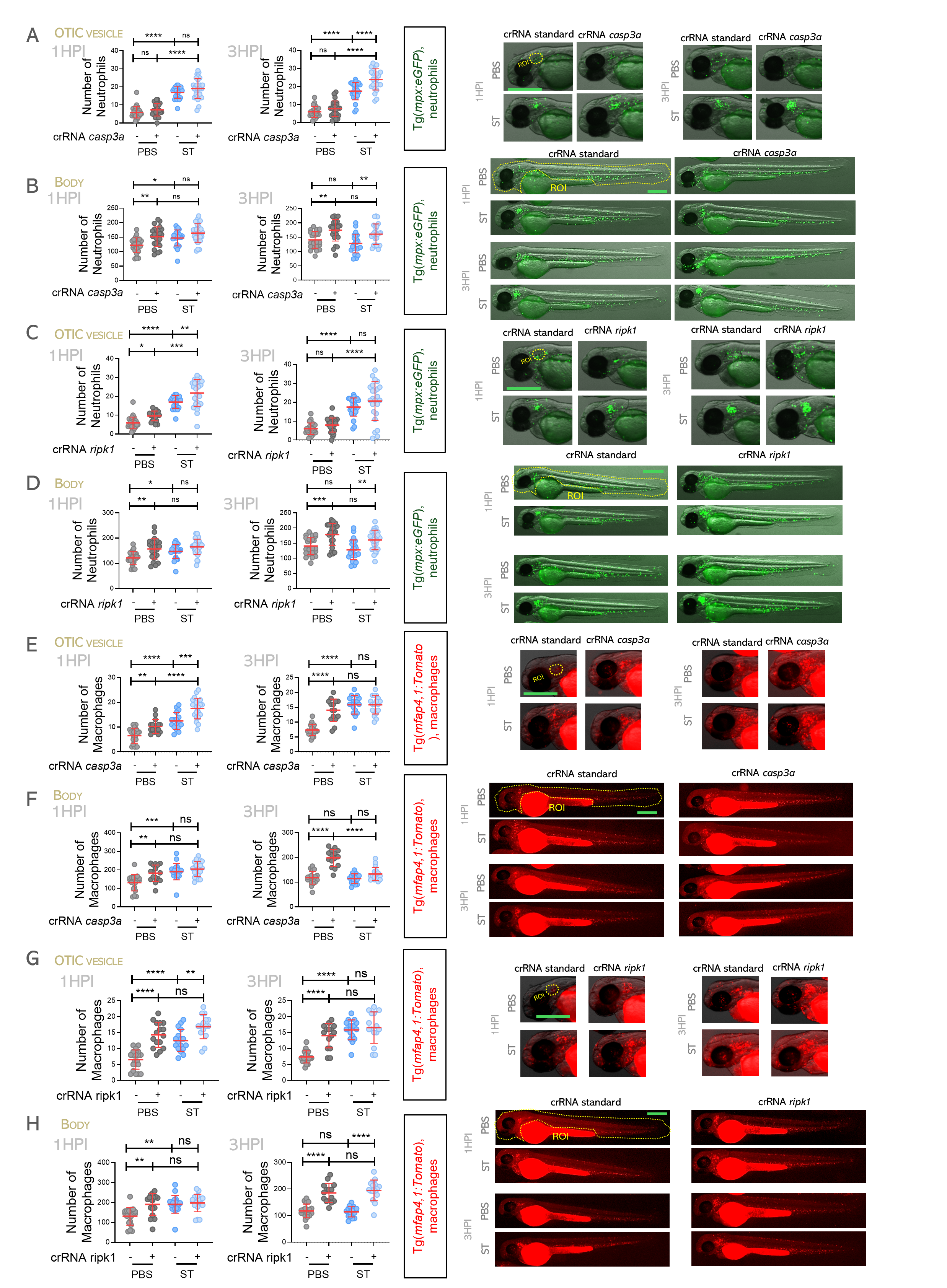

Supplement: Supplementary file 2 — Figure S2 [file 41419_2025_8291_MOESM2_ESM.tif]

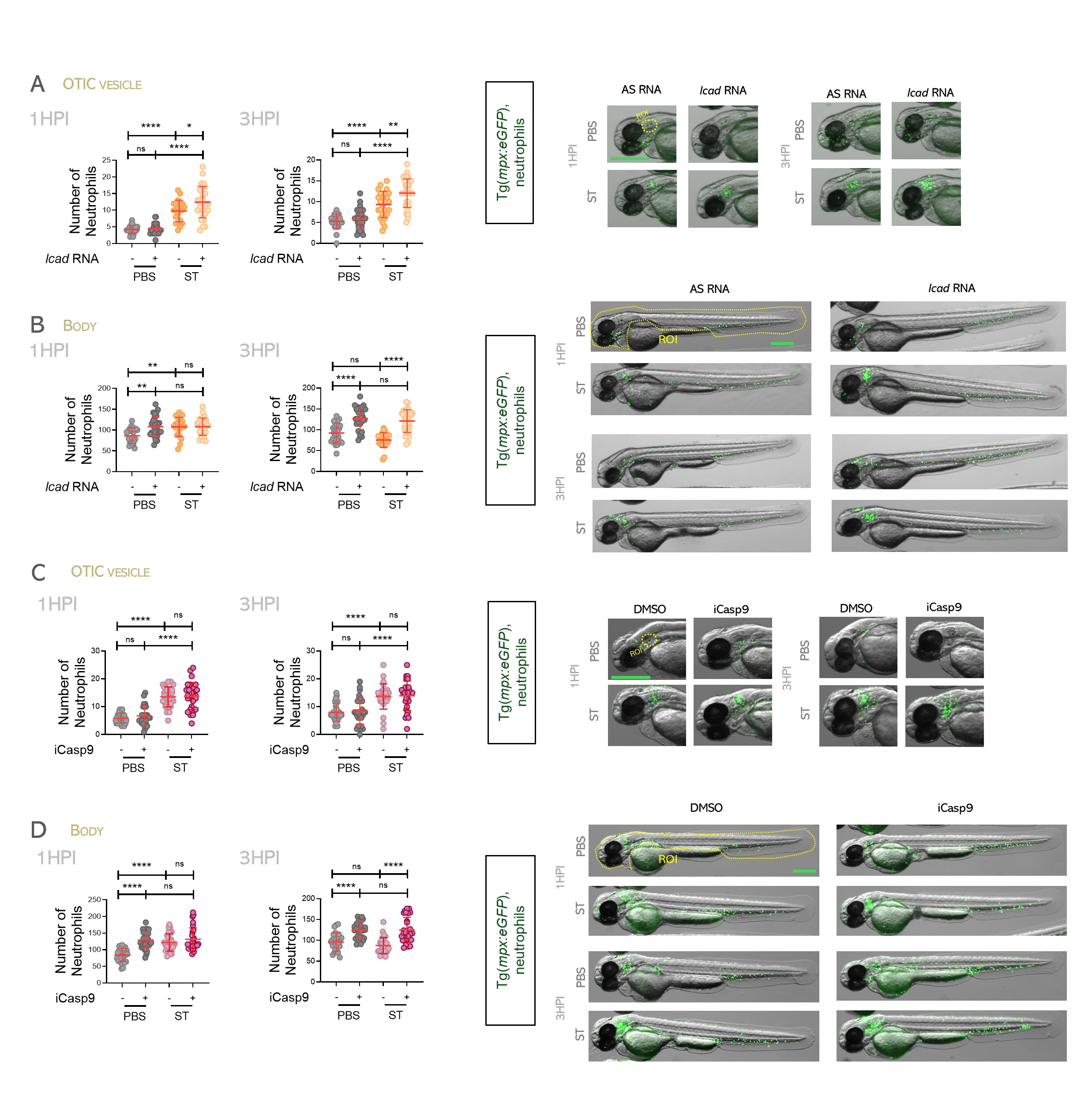

Supplement: Supplementary file 3 — Figure S3 [file 41419_2025_8291_MOESM3_ESM.tif]
