# Supplementary material for: Programmed cell death pathways coordinate neutrophil and macrophage clearance in zebrafish and are differentially exploited by Salmonella Typhimurium
Source: Cell Death Dis. 2025 Dec 8;17(1):86. doi: 10.1038/s41419-025-08291-8 (PMC12830592; doi:10.1038/s41419-025-08291-8)
Supplement: Supplementary file 4 — Supplementary Figures Legends [file 41419_2025_8291_MOESM4_ESM.docx]

**Figure S1**. One-cell stage embryos were injected with *nlrp3* gRNAs/Cas9 complexes, then 2 dpf embryos were infected in the otic vesicle with wild-type strain of ST, controls were injected with PBS. A and B, TUNEL assay and immunohistochemistry staining was performed. A, number of TUNEL positive cells in the head at 4 hpi. B, TUNEL positive neutrophil count at 2 and 4 hpi in the head or whole body. Each dot represents one individual, and the means ± SEM for each group is also shown. P values were calculated using one-way analysis of variance (ANOVA) and Tukey multiple range test. ns, not significant; *P ≤ 0.05, **P ≤ 0.01, ***P ≤ 0.001, and ****P ≤ 0.0001. The regions of interest (ROI) used for quantification in all experiments are indicated in the representative images. Bars: 500 µm.

**Figure S2**. One-cell stage embryos were injected with standard, *casp3a* (A, B, E, F) or *ripk1* (C, D, G, H) gRNAs/Cas9 complexes, then 2 dpf embryos were infected in the otic vesicle with wild-type strain of ST, controls were injected with PBS (A-H). A-D, neutrophil recruitment to otic vesicle or total count was followed up at 1 and 3 hpi. E-H, macrophage recruitment to otic vesicle or total count was followed up at 1 and 3 hpi. Each dot represents one individual, and the means ± SEM for each group is also shown. P values were calculated using one-way analysis of variance (ANOVA) and Tukey multiple range test. ns, not significant; *P ≤ 0.05, **P ≤ 0.01, ***P ≤ 0.001, and ****P ≤ 0.0001. The regions of interest (ROI) used for quantification in all experiments are indicated in the representative images. Bars: 500 µm.

**Figure S3**. One-cell stage embryos were injected with *icad* or control mRNA (A, B) or 2 dpf control embryos were treated with the inhibitor of Caspase-9 for 1h (iCasp9 - Ac-LEHD-CMK) (C, D), then 2 dpf embryos were infected in the otic vesicle with wild-type strain of ST, controls were injected with PBS (A-D). A-D, neutrophil recruitment to otic vesicle or total count was followed up at 1 and 3 hpi. Each dot represents one individual, and the means ± SEM for each group is also shown. P values were calculated using one-way analysis of variance (ANOVA) and Tukey multiple range test. ns, not significant; *P ≤ 0.05, **P ≤ 0.01, ***P ≤ 0.001, and ****P ≤ 0.0001. The regions of interest (ROI) used for quantification in all experiments are indicated in the representative images. Bars: 500 µm.
