# Supplementary material for: Programmed cell death pathways coordinate neutrophil and macrophage clearance in zebrafish and are differentially exploited by Salmonella Typhimurium
Source: Cell Death Dis. 2025 Dec 8;17(1):86. doi: 10.1038/s41419-025-08291-8 (PMC12830592; doi:10.1038/s41419-025-08291-8)
Supplement: Supplementary file 5 — Table S1 [file 41419_2025_8291_MOESM5_ESM.docx]

**Table S1.** gRNA used in this study. The gene symbols followed the Zebrafish Nomenclature Guidelines (<http://zfin.org/zf_info/nomen.html>).

| **Gene** | **ENA or ENSEMBL accesion number** | **Name** | **Sequence (5’**→**3’)** |
| --- | --- | --- | --- |
| *gsdmea* | ENSDARG00000086762 | CD.Cas9.PQCQ3167.AA | CATCCCACCGGACACTGTGT |
| *gsdmeb* | ENSDARG00000040485 | Dr.Cas9.DFNA5B.1.AO | GCAGACACGGAACGCAAAGA |
| *nlrp1* | ENSDARG00000088423 | CD.Cas9.JTJN2987.AA | TCACAGAAGACTCAACTAGC |
| *nlrp3* | ENSDARG00000078620 | CD.Cas9.BNQV6690.AA | GGACATCGTCCACTGAACTA |
| *casp3a* | ENSDARG00000017905 | Dr.Cas9.CASP3A.1.AE | AGCTTGAACTACCCCAACAT |
| *ripk1* | ENSG00000137275 | Dr.Cas9.RIPK1L.1.AD | CGGTTCCAATATCTGTGAAA |
