# Supplementary material for: Programmed cell death pathways coordinate neutrophil and macrophage clearance in zebrafish and are differentially exploited by Salmonella Typhimurium
Source: Cell Death Dis. 2025 Dec 8;17(1):86. doi: 10.1038/s41419-025-08291-8 (PMC12830592; doi:10.1038/s41419-025-08291-8)
Supplement: Supplementary file 6 — Table S2 [file 41419_2025_8291_MOESM6_ESM.docx]

**Table S2.** Primers used in this study. The gene symbols followed the Zebrafish Nomenclature Guidelines (<http://zfin.org/zf_info/nomen.html>).

| **Gene**  **(ENSEMBL accesion number)** | **Name** | **Sequence (5’**→**3’)** | **Use** |
| --- | --- | --- | --- |
| *cxcl8a*  (ENSDARG00000104795) | F | GTCGCTGCATTGAAACAGAA | RT-qPCR for determination of gene transcript levels |
|  | R | CTTAACCCATGGAGCAGAGG |  |
| *rps11*  (ENSDARG00000053058) | F | ACAGAAATGCCCCTTCACTG |  |
|  | R | GCCTCTTCTCAAAACGGTTG |  |
| *il1b*  (ENSDARG00000098700) | F | GCCTGTGTGTTTGGGAATCT |  |
|  | R | TGATAAACCAACCGGGACA |  |
| *tnfa*  (ENSDARG00000009511) | F | GCGCTTTTCTGAATCCTACG |  |
|  | R | TGCCCAGTCTGTCTCCTTCT |  |
| *nfkb1*  (ENSDARG00000105261) | F | TTCTTCTTGGTCACGTGCAG |  |
|  | R | ACTCTCAGCATCCGCATCTT |  |
| *gsdmea*  (ENSDARG00000086762) | F | ACTGGACCTTAAACACTCTTTGAT | PCR for determination of genetic edition efficiency |
|  | R | ataccgtctgtctgtcagtgc |  |
| *gsdmeb*  (ENSDARG00000040485) | F | GCTGGATTTGCAGAAGCTCTTACATG |  |
|  | R | AACCACCCTTGCTTACAGCG |  |
| *nlrp3*  (ENSDARG00000078620) | F | GGGGGAAGGACAAGGGAAAC |  |
|  | R | GACATTCCCTCCACCCTCAC |  |
| *casp3a*  (ENSDARG00000017905) | F | ACCCACAGAAATCATGTGCAATC |  |
|  | R | ATCAGCCATACTTTTGAAACCCTT |  |
| *ripk1*  (ENSG00000137275) | F | TCGCGATCTGCAACACCTAGA |  |
|  | R | TCGTCCTCGACTTTATACTGA |  |
